# Book Reviews

**Published:** 1855-06

**Authors:** 


### HEALTH, DISEASE, AND LONGEVITY.

By Lionel John Beale, M.R.C.S. London: 1854.

The object of this excellent work is to expound the general laws of health in a popular manner, and to show that health is much more under the individual will than is commonly supposed.

After noticing the rising importance of hygiene, and showing the means of carrying it into effect, with the results of attention or

---

non-attention to its laws, the author gives a comprehensive view of the functions of the organs of digestion, respiration, &c. The substances used by these organs, in the digestive and respiratory processes, may be arranged in four classes.

The aqueous; constituting four-fifths of the blood, in addition to the many offices it has to perform in the system.

The oleaginous and saccharine; used in the production of animal heat; or, if in excess, laid up in fat-cells.

The albuminous; supporting the nervous and muscular systems, and the tissues composing the various organs of the animal body.

The saline; entering into the composition of bone and of the blood.

These four classes in due combination compose the blood. The most suitable diet, then, is that in which the materials of life are combined in the right proportion. Deficiency or excess in any of these four principles is attended with corresponding evils to the healthy action of organs.

To form blood and to supply materials for the animal heat are the chief objects of the process of digestion. To effect the first of these we require the nitrogenous substances, whilst the latter is attained by the non-nitrogenous. Mr. Beale explains how each of these is acted upon by the digestive and respiratory organs, shewing the conversion of food into starch, of starch into sugar, and of sugar into fat. This fat, slowly oxidized by atmospheric and other influences, supplies the fuel for the animal heat.

The influence of climate, and of the age, habits and occupation of the individual, is shewn to be very great as regards the quantity and quality of food. Children, and persons much in the open air, require, generally, more food than adults, and persons of sedentary habits. The animal heat is greater in the former, consequently the amount of food (or fuel) oxidized must be greater; whereas with the latter the demand for food is smaller, from the slower combustion, and the consequent slower decrease of the tissues of the body.

Having treated of the various organic functions of the body, and the substances submitted to their action, with the manner in which they are assimilated, for the renovation of the system—the author shows what is necessary to the "foundation of a sound constitution and the reparation of a broken one."

"When people become valetudinarians, they begin to attend to the laws of health." Unsound health, both of mind and of body, is easily traceable to imperfect education; for our bodies are often disordered by absurd indulgences, and our minds immersed in prejudices, from our very infancy; and the effects of these are seldom obliterated. Parents, then, should be able to impart the laws of health to their children; and the study of these laws ought in a perfect system of education to find a prominent place. We see the good results of attention to the improvement of a breed of horses or sheep, and why may not the same effects be attained in the human being? As the case is at present, a child is born, and handed over to the nurse, who not unfrequently possesses a large amount of prejudice on things "always done," and an equally large amount of ignorance as to what ought to be done.

The principles necessary to the foundation of a sound constitution, are few, and they demand attention from the earliest period. The most important of these principles are,—strict cleanliness; good simple food taken in moderate quantities and at regular intervals; pure air; exercise, both physical and mental; easy fitting clothes suited to the season; and a proper amount of rest. Too often are these rules neglected. Many a healthy child cries on being washed; sweetmeats or pastry are given to quiet him; thus the healthy action of the skin is destroyed, and disease of the stomach is induced at the same time. Frequently also, children, well formed at birth, become narrow and pigeon-breasted in a few months, from bandaging and other mismanagement. Above all, the training of the mental faculties should not be neglected, but habits of close observation, and the power of fixing the mind, should be cultivated as the faculties begin to develope themselves.

The rules laid down for the reparation and re-establishment of an unsound constitution are few and simple. With many children disease is inherent; in others it is gradually induced by injudicious treatment; but whatever the cause of disease, its eradication is much promoted by attention to the laws of health. The food of invalids should consist simply of such things as are essential to the nourishment of the body, and these should be taken with regularity and moderation. Baths, friction, and exercise, also play an important part in the cure of many diseases. Diseases of the nervous system are of frequent occurrence. Many causes, some of them diametrically opposite, contribute to this list,—as idleness, extreme mental exertion, joy, sorrow, fear, &c. In all cases, much may be done for the relief of the sufferer by a judicious combination of mental and physical remedies.

The last part of the work treats of longevity. We have a large number of examples of old age from various countries, with a short notice of the mode of living of many of them, and from them some general principles are deduced as to the best modes of prolonging life. Great Britain, Sweden, and Denmark, record many cases of old age: hence it appears that a cold and rather moist climate is conducive to longevity. Perhaps spare diet also has something to do with this fact. Residence in cities tends to shorten life. In Liverpool one half the children born, die before the age of five years: very nearly the same obtains in Manchester and Birmingham. In London one half of the children born live to the age of twenty. In Liverpool only fifty-three persons out of a thousand live beyond the age of seventy, whilst in Westmoreland the population is 203 in a thousand.

There is no universal rule for the attainment of long life. In the list of examples given, we find persons of very different habits and constitutions; some used vegetable food; some animal; some drank water, others used wine or beer: but *very few* of them were spirit drinkers, and both town and country contribute to the list. To sum up:—Attention to the laws of health, regularity, moderation in all things, cheerfulness, due exertions both of mind and body, that acquiescence with the position in life in which circumstances have placed the individual, and making the best of every event that may occur, appear to be among the more important elements of longevity, a healthy life, and a mature old age.

---

### HUMAN LONGEVITY AND THE AMOUNT OF LIFE ON THE GLOBE.

By P. Flourens. Translated from the Second Edition by Charles Martel. 12mo, pp. 198. London: 1855.

This book has already gained a great and, in many respects, a deserved popularity. It is a philosophical, manly written production; a little too dogmatic here and there, but learned, and, at the same time, most original. The object of the author is mainly to show, that the natural duration of human life is not less than one hundred years. He is a great admirer of Cornaro, whom he holds up as a model man in regard to the observance of the laws of health. This Cornaro lived to be ninety-eight, having, as he believed, prolonged his days to this late period by a peculiar mode of living: in which he avoided all excitement, courted seclusion, fulfilled all natural laws according to the best of his belief, and, above all things, avoided laborious pursuits both mental and bodily. If some other man were to live like Cornaro, he might, perhaps, live as long. But what philosopher shall devise the social problem, that shall enable us all to become Cornaros? Cornaro made for himself, in fact, a distinct life in respect to the great world around him; and if all were to follow his plan, granting that they could do so, it is questionable whether the general result would be the same as the particular:

> "For some must watch, and some must sleep,
> Thus runs the world away."

Ramazzini, an eminent physician and commentator on Cornaro, makes some able remarks, which M. Flourens quotes, but evidently not with a relish.

"It would be too severe," says Ramazzini, speaking of Cornaro's rules, "to prescribe such rules to persons who enjoy perfect health; it would not even be good for the public. Let old men do so after they have passed the best part of their lives in the service of the republic; but it is not just to include young men in these observations. How will they be able to serve their prince and country, either in embassies or armies, where the fatigue of marches is required? How will a doctor be able to visit his patients every day? How will a lawyer be able to discharge his duties? If any man asked me what food to use, in what quantity, and at what times he ought to take it to sustain his health, I should refer him to his stomach, which is doubtless more capable than any one of giving him good advice on that subject."

These remarks of Ramazzini embrace the soundest health rules. It is no object of the true sanitary reformer to invent impossible and mere ideal laws, suitable at most to single individuals peculiarly placed and constituted; it is his business rather to point out such general measures as shall enable the citizen of the world to live in the world, with all its jostlings, oddities, and changes, with the least possible exposure to the encroachments of time and disease.

M. Flourens argues that the natural duration of life in every animal is proportioned to the duration of the time of its growth. An animal lives about five times the period that it took to complete its growth. Man attains his full growth in twenty years; *ergo*, he can live five times that period, or one hundred years, and this is the natural term of life.

With Buffon, M. Flourens is inclined to the probable and grand idea, that "the total quantity of life on the globe is always the same, and that death, which seems to destroy everything, destroys nothing of that primary life common to every species of organized beings; that like all other subordinate and inferior powers, death attacks only individuals, only strikes the surface, can effect nothing upon matter, and does no harm to nature, which only shines the brighter, to show itself independent of death and time,—to exercise at every moment its ever active power,—to manifest its plenitude by its fecundity, and to make of the universe a theatre always full, a spectacle ever new, by the perpetual reproduction of beings."

We have given but a very faint reflection of M. Flourens' work; but sufficient to show that it emanates from a mind charged with bold conceptions and broad views regarding the planet earth and its various inhabitants.

---

REPORT ON THE MORTALITY AND PUBLIC HEALTH OF OXFORD DURING THE YEARS 1849-50: Oxford: 1854.

QUARTERLY REPORTS ON THE SICKNESS AND MORTALITY IN THE BOROUGH OF SWANSEA: from the 1st of October, 1853, and the 1st of July, 1854. Swansea: 1854.

THE Report on the Mortality and Public Health of Oxford was drawn up by Dr. W. A. Greenhill (now of Hastings), and Mr. Thomas Allen. We have here monthly and quarterly reports of mortality, published by permission of the Registrar-General. The first appendix contains information on the marriages, births, and deaths in Oxford. In the second appendix we meet with some valuable data regarding the influence of impure water in propagating cholera. One part is worthy of notice—that the parish of St. Clement was severely visited by cholera in 1832, while the epidemic was only slightly felt there in 1849; and this appears attributable to the supply of water having in the interval been brought from a distance, instead of from the river in the vicinity.

The Reports on the Sickness and Mortality in Swansea, which were drawn up by Mr. W. H. Michael, the medical officer of health for the borough, contain some remarkable illustrations of the evil effects of inattention to sanitary rules.

Reports of this kind are most valuable, and we hope to have many opportunities of noticing similar documents.

---

### TRANSFUSION OF MILK, AS PRACTISED IN CHOLERA, AT THE CHOLERA SHEDS, TORONTO, UPPER CANADA.

By JAMES BOVELL, M.D. Pamphlet, pp. 16. Toronto.

THIS *brochure* was read before the Canadian Institute. It contains the history of six cases of cholera, treated during their last stages by the transfusion into the veins of freshly drawn cow's milk. Two of these cases recovered. There can be no doubt that this kind of treatment is that to which sound and philosophical pathology points most directly. Milk is possibly the best fluid that can be injected into a vein, and Dr. Bovell deserves great credit for having thrown out and practised the suggestion. Two successful cases are not sufficient, however, to establish a practical point; and we almost fear, from the small quantities of milk employed, (twelve ounces in one case and eight in another,) that some agents were at work which led to the recovery, over and above the treatment thus carried out.

It is fair to observe, that although Dr. Bovell was the first to offer the suggestion of injecting milk into the veins of cholera patients, Dr. W. Bird Herapath, of Bristol, threw out the same suggestion on September the 1st, 1854, in the *Association Medical Journal;* and this quite as an independent and original idea.

---

### THE USE OF VEGETABLE AND MINERAL ACIDS IN THE TREATMENT OF CHOLERA AND OTHER BOWEL DISORDERS.

By J. H. TUCKER, Hon. Sec. of the Epidemiological Society. London.

As the founder of the Epidemiological Society, and as an investigator of subjects bearing on epidemics, Mr. Tucker is well known and appreciated in the medical profession. In the work before us, he is intent to show that the use of the vegetable and mineral acids is both preventive and curative of choleraic disorders. The vegetable acids have long been known as useful agents of the materia medica, and Mr. Tucker has laboured hard and learnedly in establishing his peculiar views. Mr. Tucker dwells especially on the virtues of cider, which beverage, according to him, is a prophylactic of the first order. Without at all doubting the importance of the vegetable and mineral acids as useful medicines, we cannot believe that our author has proved his point. He has, however, raised an interesting question, which observers of epidemics will do well to bear in mind.

